# An Updated Review on Silver Nanoparticles in Biomedicine

**DOI:** 10.3390/nano10112318

**Published:** 2020-11-23

**Authors:** Oana Gherasim, Rebecca Alexandra Puiu, Alexandra Cătălina Bîrcă, Alexandra-Cristina Burdușel, Alexandru Mihai Grumezescu

**Affiliations:** 1 Department of Science and Engineering of Oxide Materials and Nanomaterials, University Politehnica of Bucharest, 1-7 Gheorghe Polizu Street, 011061 Bucharest, Romania; oana.fufa@gmail.com (O.G.); rebecca_alexandra92@yahoo.com (R.A.P.); ada_birca@yahoo.com (A.C.B.); alexandra_burdu@yahoo.com.sg (A.-C.B.); 2 Lasers Department, National Institute for Lasers, Plasma and Radiation Physics, 409 Atomistilor Street, 077125 Magurele, Romania; 3 Research Institute of the University of Bucharest—ICUB, University of Bucharest, 90-92 Panduri Road, 050657 Bucharest, Romania

**Keywords:** antimicrobial therapy, cancer therapy, tissue engineering, wound care

## Abstract

Silver nanoparticles (AgNPs) represent one of the most explored categories of nanomaterials for new and improved biomaterials and biotechnologies, with impressive use in the pharmaceutical and cosmetic industry, anti-infective therapy and wound care, food and the textile industry. Their extensive and versatile applicability relies on the genuine and easy-tunable properties of nanosilver, including remarkable physicochemical behavior, exceptional antimicrobial efficiency, anti-inflammatory action and antitumor activity. Besides commercially available and clinically safe AgNPs-based products, a substantial number of recent studies assessed the applicability of nanosilver as therapeutic agents in augmented and alternative strategies for cancer therapy, sensing and diagnosis platforms, restorative and regenerative biomaterials. Given the beneficial interactions of AgNPs with living structures and their nontoxic effects on healthy human cells, they represent an accurate candidate for various biomedical products. In the present review, the most important and recent applications of AgNPs in biomedical products and biomedicine are considered.

## 1. Introduction

Nanotechnology and nanoscience represent important fields for the progress of modern society, especially given the incessant efforts and impressive achievements in alternative nano-based therapies [1,2]. A particular interest was oriented towards the revaluation and biofunctional assessment of metallic nanoparticles for biotechnology and biomedicine, especially thanks to their unique physical, chemical and biological features [3,4,5]. Specifically, biocompatible nanoparticles with superior physicochemical properties, suitable biomechanical behavior and tunable therapeutic efficiency can be successfully obtained [6,7]. Thanks to their genuine size-related characteristics, nanoparticles are distinguished as the most versatile candidates for biotechnological and biomedical applications, being considered the star technology of the 21st century [8,9].

Among zero-dimensional nanomaterials, silver nanoparticles (AgNPs) represent one of the most explored and promising candidates for unconventional and performant applications in the contemporary world, with formidable results being reported in pharmaceutical sciences [10,11,12], cosmetic products [13,14], anti-infective coatings [15,16] and wound dressings [17,18], antimicrobial textiles [19,20,21] and food packages [22,23,24]. The particular interest of AgNPs in biomedical applications mainly relies on their excellent and extensive antimicrobial properties, limited anti-pathogenic resistance and impressive efficiency against multidrug-resistant microorganisms [25,26,27].

AgNPs with tunable physicochemical characteristics and versatile functionality can be obtained by various *top-down* (mainly, evaporation-condensation processes of bulk silver) [28,29] and *bottom-up* (mainly, electrochemical processes of metallic salts) processing methods [30,31]. Special attention was oriented on the inexpensive and environmentally-friendly synthesis of AgNPs, which either considers the revaluation of plant-derived reducing and antioxidant phytochemicals [32,33] or the microorganism-mediated bioreduction mechanisms [34,35].

Given their intrinsic antimicrobial efficiency, silver-based compounds and materials were used for many centuries in day-to-day applications [36,37,38]. Their versatility and excellent biofunctionality enabled the development and clinical implementation of several human-safe commercial products, as summarized in Table 1.

**Figure 1:**
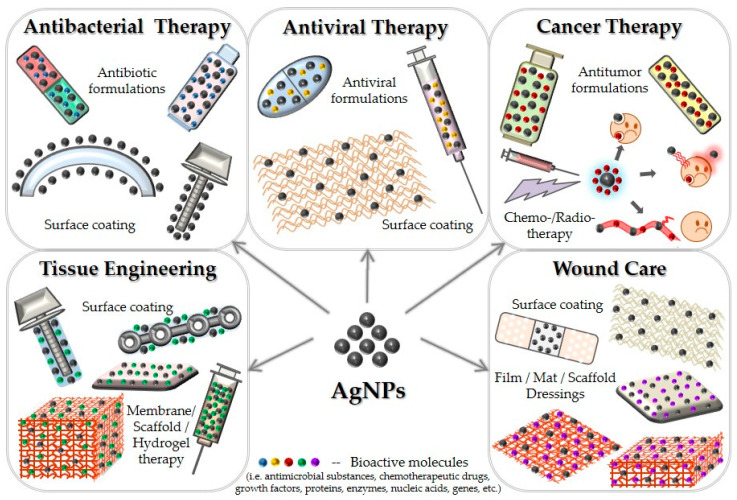
Applications of silver nanoparticles (AgNPs) in biomedicine.

Besides commercial products, an impressive number of preclinical studies reported the successful implications of AgNPs in the development of effective and performance-enhanced unconventional therapeutic strategies. A schematic representation of the most explored therapeutic applications of AgNPs in modern healthcare practice is included in Figure 1. The present paper aimsto survey the most recent biomedical applications of nanosilver-based formulations.

## 2. Toxicity of Silver Nanoparticles

Nanosilver is of great interest for modern and personalized biomedical uses, especially since their interactions with living structures may lead to biochemical modifications in cytoskeleton organization and molecule adhesion [39,40], as well as in cell proliferation [41,42]. In a similar way to their intrinsic anti-pathogenic effects, the AgNPs-mediated toxicity in mammalian cells may be induced by a different mechanism, such as: (i) disruption in energy-dependent cellular events and impairment in DNA replication, caused by the uptake of free silver ions; (ii) formation of reactive oxygen species and free radicals; and (iii) local damage of cellular membrane due to direct interactions with AgNPs [43,44].

Biosynthesized nanosilver coated with cetyltrimethylammonium bromide (CTAB) surfactant and polyethylene glycol (PEG) polymer showed time- and dose-dependent effects on erythrocytes with increasing the concentration of coating agent, but exhibited nonhemolytic activity at CTAB and PEG concentrations below 100 µg/mL, being thus considered blood compatible [45]. Highly stable and conductive nanosilver stabilized with a polymer coating of polyvinyl alcohol (PVA)—PEG and poly(3-aminophenyl boronic acid) also proved compatible for human red blood cells. The nanocomposite exhibited nontoxic effects on normal human cell line at bactericidal concentrations [46].

Biosynthesized AgNPs coated with chitosan (CS) exhibited enhanced anticoagulant activity in an animal model, as evidenced by the dose-dependent variation of blood parameters. Also, the treatment with nanoparticles determined increased antiplatelet and thrombolytic effects, as well as low cytotoxicity in different study models [47].

The size-dependent phagocytic internalization of AgNPs proved responsible for cytotoxic effects on macrophages. Significantly increased levels of reactive oxygen species (ROS) and interleukin were reported when treating the cells with 4 nm sized nanosilver, whereas the 20 and 70 nm nanoparticles led to more reduced or even insignificant cytotoxicity [48]. As evidenced by complex structural and functional tests and proteomic assays, macrophages treated with silver nanoparticles stabilized with polyvinylpyrrolidone (PVP) exhibited cellular homeostasis recovery within three days after acute exposure [49]. Moreover, highly antibacterial and anti-biofilm AgNPs coated with bacteria-isolated protein showed no toxic effects against primary macrophage cultures and different vital organs [50].

A complex study proved that AgNPs stabilized with PVP have dose-dependent toxic effects on murine dendritic cells, but the effects were significantly increased against cancerous cells [51]. Particles of 10 nm exhibited toxicity on neural stem cells, regardless the type and charge of a surface coating. Oxidative events, as well as ROS-mediated inflammatory response and DNA damage, caused either apoptotic or necrotic cell death [52]. The size-dependent neurotoxicity of PVP-coated nanosilver was reported by Zhang et al., as 20 nm particles induced increased intracellular silver accumulation and caused significant cytoskeleton modifications and dopamine efflux alterations, in comparison with 70 nm particles [53].

Negatively charged AgNPs biosynthesized with sorrel flower extract determined dose-dependent toxicity in human endothelial cells, causing ROS-mediated apoptosis, as well as cellular morphological and functional impairment. The as-obtained particles induced cell malformations, death and senescence in a zebrafish model due to severe oxidative stress [54]. Also, Jalilian and coworkers reported the dosage-dependent cytotoxicity of nanosilver on normal fibroblasts. The same nanoparticle concentrations induced higher cytotoxic effects against malignant cells [55].

No toxic or genotoxic effects were identified in fibroblast cultures treated with nanoparticles coated with silica (SiO_2_) [56]. In a comparative study, Verkhovskii et al. reported that highly stable AgNPs coated with PVA and sodium carboxymethyl cellulose (CMC) are safe for dermal fibroblasts, while nanoparticles coated with sodium dodecyl sulfate and sodium oleate proved cytotoxic [57]. The viability of human lung fibroblasts was minimally reduced when treated with AgNPs in concentrations up to 100 µg/mL or 2.5 mg/mL, obtained with Ayurvedic medicinal herb extract [58] or oxidized amylose/curcumin complex [59], respectively. The biosynthesized nanoparticles also exhibited dose-dependent antioxidant activity while showing enhanced antibacterial effects. As well, bactericidal concentrations of lecithin-modified montmorillonite (MMT) embedded with nanosilver showed no toxic effects on normal fibroblasts [60]. Antibacterial composites of high-density polyethylene and AgNPs-decorated MMT exhibited superior cytocompatibility with human erythrocytes and dermal fibroblasts and caused no morphological alterations in the skin tissue of rats after 21 days of exposure [61].

Concentrations lower than 10 µM of highly stable PEG-coated nanosilver proved safe for human keratinocytes, while the higher concentration of 50 µM was associated with intense cell mortality [62]. Graf and coworkers reported no preferential shape-related cellular internalization of AgNPs by keratinocytes, while mesenchymal stem cells exhibited preferential uptake of PVP-stabilized silver nanoprisms, in comparison with silver nanospheres [63]. Reduced levels of cytotoxicity and genotoxicity on human keratinocytes were also reported in the case of nanoparticles biosynthesized by *Trichoderma harzianum* cultivated with or without pathogenic fungal enzymes [64]. Fungi-mediated bioreduced AgNPs exhibited high antimicrobial efficiency, but showed nontoxic effects against normal human melanocytes for concentrations up to 6 µg/mL [65].

Huy and coworkers reported that silver nanoparticles synthesized by the electrochemical method were nontoxic on striated muscle cell cultures for concentrations up to 100 ppm but displayed strong biocidal effects against enveloped viruses [66]. Mild lung inflammatory infiltrate was observed after the pulmonary exposure of albino mice to AgNPs coated with PVP or citrate. Consequently, dose-dependent effects were evidenced at the cardiovascular level, such as the increase of proinflammatory cytokines and antioxidants, cardiac oxidative stress, DNA damage and apoptosis of cardiac cells, prothrombotic events, and coagulation alterations [67].

Different studies from recent years investigated the toxicity of nanosilver with respect to human tissues and demonstrated their applicability as safe therapeutic agents for pharmaceutical products [68,69]. An important fact is that the toxic effects exhibited by AgNPs are strongly influenced by their morphology and dimension. For example, cytoplasm and cellular organelles are more prone to be affected by smaller particles than bigger particles [70,71].

The main mechanism responsible for AgNPs-related cytotoxicity is the oxidative stress induced by the excessive production of ROS, which can cause structural and functional impairments in proteins, lipids and DNA, resulting in cellular alteration and even cell death [72,73]. More than that, due to their nanodimension and reactive surface chemistry, AgNPs are captured and internalized by the cells [74]. Their intracellular agglomeration can severely damage cellular constituents (cell wall, cytoplasm), as well as vital organelles (mitochondria) and essential macromolecules (proteins, enzymes, DNA) [75,76]. Also, another possible mechanism for nanosilver toxicity is cellular apoptosis [77,78].

Hu and coworkers reported that silver nanoparticles affect the global differentiation of human embryonic stem cells. In particular, dose-dependent effects were evidenced during the differentiation and function of hepatocytes and cardiomyocytes [79]. Using albino mice, Jarrar et al. proved that hepatoprotective agents or coatings are mandatory for the safe administration and suitable drug metabolism of AgNPs-based systems, as citrate-coated nanosilver (20 ± 5 nm size) induced significant downregulation in the gene expression of hepatic drug-metabolizing enzymes, causing important hepatic inflammation [80].

Green synthesized AgNPs altered the levels of glucose and hepatic enzymes in an animal model, but did not induce morphological modifications. The beneficial conjunction between metallic nanoparticles and common dogwood extract resulted in diminished oxidative stress and enhanced antioxidant and anti-inflammatory effects [81]. The functional alteration of liver and kidney was also reported after the inoculation of CS-coated bioreduced nanosilver in rats. Moreover, the proposed AgNPs proved able to cross the placenta and induced minimal toxicity in fetuses. Contrariwise, time-dependent severe fetal hepatotoxicity was evidenced in the case of uncoated nanoparticles inoculation [82].

Other studies reported the importance of surface coating on the bioavailability and toxicity of nanosilver. AgNPs modified with silicate and PVP neutral coatings induced less inflammatory effects and genotoxicity than negatively charged citrate and positively charged branched polyethylenimine (PEI) coatings [83]. In addition, Zucker and coworkers showed that 80 nm AgNPs coated with branched PEI were internalized and accumulated at a greater rate by epithelial cells, in comparison with nanoparticles coated with citrate, PEG, or PVP, causing significant mitochondria damage [84].

Following the subacute exposure of rats to AgNPs, Docea and coworkers evidenced significant antioxidant effects in the case of particles coated with ethylene glycol (EG). For the same doses, PVP-EG-coated AgNPs resulted in important pro-oxidant effects, as indicated by the induction of protein oxidation and decrease of glutathione levels [85]. After subcutaneous implantation in rats, colloidal nanosilver determined important subacute inflammatory response of connective tissue but demonstrated long-term biocompatibility, as evidenced after 60 days of exposure [86].

## 3. Silver Nanoparticles for Antibacterial Applications

An alarming phenomenon of current healthcare practice is the occurrence of many drug-resistant microorganisms, which leads to ineffective conventional monotherapy. Bacterial infections and their related complications represent a major and frequent cause of death [87,88]. With the aim to overcome the limitations that occurred due to drug-resistant pathogens, worldwide researches focused on the investigation of antibacterial resistance mechanisms, as well as on the development and optimization of unconventional and effective antibacterial strategies.

A concerning number of bacterial strains developed or enlarged their resistance to conventional antibiotherapy, especially due to their versatile mechanisms to adapt to the action of drugs and to the excessive usage of antibiotics [89,90,91]. The clinical implications related to infections caused by drug-resistant bacteria and the particular complications related to biofilm-embedded pathogens led to the necessity to develop new and effective bactericidal products [92,93,94]. In particular, nanomaterial-based formulations represent a feasible choice for modern and therapeutic-enhanced antibacterial agents.

Silver-based compounds have been used as antimicrobial agents for thousands of years, proving the ability to go through biological membranes and to exhibit local or systemic effects, thus being used for different treatments, including dental and digestive pathologies, wounds and burns healing [36,95]. Despite their remarkable therapeutic properties, the weakness of such compounds is related to their toxicity on human cells, which occur at higher concentrations. In addition, if prolonged treatment with silver-based compounds is applied, their accumulation in the organism may lead to vital organs’ impairment and skin discoloration (argyria) [96,97]. Therefore, in order to overcome cytotoxicity, products containing silver compounds and nanoparticles require very low metallic concentrations and suitable delivery systems.

The effects of biocompatible systems based on nanosilver for novel antimicrobial agents were assessed against various planktonic and sessile strains relevant to current clinical practice. In particular, a plethora of research studies investigated the effects of nanosilver-based biomaterials against *Escherichia coli* (*E. coli*) and *Staphylococcus aureus* (*S. aureus*), as representative (respectively) Gram-negative and Gram-positive pathogens responsible for community-transmitted and hospital-acquired infectious diseases.

In comparison with *S. aureus*, the increased sensitivity of *E. coli* to AgNPs bactericidal activity was related to the much thinner peptidoglycan layer and the outer liposaccharide portion within the cell wall, which can modulate bacterial membrane destabilization, cell penetration and leakage of intracellular organelles and macromolecules [98,99]. Due to high surface reactivity, strong interactions formed between nanosilver and bacterial membranes containing phosphorous and sulfur determined significant growth inhibition of both strains [100,101]. The antibacterial effects of AgNPs were also related to cellular stress induced by the alteration of energy-dependent processes mediated by adenosine triphosphate (ATP) [102,103].

Moreover, it was demonstrated that the toxic effects exhibited by AgNPs against *E. coli* and *S. aureus* can be mediated by the release of metallic ions (Ag^+^). The cellular exposure to silver ions was associated with increased levels of highly reactive species, such as singlet oxygen, hydroxyl, superoxide, hydroperoxyl, hydrogen peroxide and nitric oxide. It was reported that the generated ROS were responsible for cellular oxidative stress, induced by lipid peroxidation, impairment of protein and enzyme metabolism, degradation of nucleic acids [104,105].

The electrostatic affinity of Ag^+^ ions for phosphate- and thiol-containing macromolecules was associated with the inactivation or denaturation of vital macromolecules (proteins, enzymes, nucleic acids), which eventually resulted in bacterial cell death [106,107].

Size-dependent antibacterial effects of AgNPs were evidenced. Due to a larger specific surface area and intense surface reactivity, smaller nanoparticles generate better contacts with cellular structures and subcellular components and are able to induce stronger Ag^+^-mediated cellular oxidative stress [108,109]. Thermo-sensitive polymer nanoparticles embedded with AgNPs exhibited size-dependent antibacterial activity against both *E. coli* and *S. aureus* strains, the effects being more pronounced in the case of the smallest particles [110]. In a similar way, ultrasmall AgNPs (1.59 nm) stabilized with thermo-sensitive copolymer showed enhanced bactericidal effects against *E. coli* and *S. aureus* than bigger nanoparticles (2.29 and 3.91 nm sizes). An enhanced cytotoxic effect was observed against the Gram-negative pathogen, as evidenced by nanosilver-mediated damage of bacterial membrane, cellular uptake and ROS generation [111]. Smaller nanoparticles (below 20 nm) were generally reported as more cytotoxic for both Gram-positive and Gram-negative bacteria [112,113].

The antibacterial efficiency of nanosilver also proved to be shape-dependent. Spherical biosynthesized nanosilver (40 nm) showed stronger bactericidal effects against *E. coli* and *S. aureus* strains in comparison with NPs with irregular shapes [114]. Due to higher specific surface area and increased ability to release metallic ions, spherical AgNPs exhibited superior antibacterial effects compared with disk and triangular plate morphologies [115]. Excellent inhibitory effects were reported for ultrasmall spherical AgNPs (2–5 nm) bioreduced with fungal extract, in comparison with particles with pentagonal or hexagonal (50–100 nm) and rectangular (40–65) shape [116]. Chemically synthesized spherical AgNPs showed more effective killing bacteria ability than rod-shaped counterparts when used against both Gram-negative and Gram-positive pathogens. It was evidenced that the antibacterial activity of nanosilver is strongly related to their microstructure, namely the presence of (1 1 1) crystallographic plane [117].

Strong bactericidal or bacteriostatic effects against *E. coli* were also reported for AgNPs biosynthesized with gum kondagogu (4.5 ± 3.1 nm) [118], *Arisaema flavum* extract (5–8 nm) [119] and *Polygonatum graminifolium* extract (3–15 nm) [120], but also for nanoparticles capped with PVP (16 ± 2 nm) [121], pectin (8–13 nm) [122] and chitosan (>20 nm) [123]. AgNPs biosynthesized with corn silk extract (10–30 nm) [124], belladonna tincture (15–20 nm) [125], thyme extract (75 nm) [126] and nanosilver bioreduced by *Bacillus subtilis* (3–20 nm) [127] and *Lactobacillus brevis* (45 nm) [128] showed pronounced antibacterial effects against *S. aureus*.

Besides *E. coli* and *S. aureus* bacterial strains, nanosilver biomaterials proved efficient against various clinically-relevant pathogens, as summarized in Table 2. A wide variety of studies reported that, following their interaction with AgNPs, microbial cells death occurs due to (i) attachment to the cell surface, followed by modification of membrane permeability, cell wall piercing, intracellular infiltration and cytoplasm leakage [129,130]; (ii) generation of highly reactive species and free radicals, followed by denaturation of microbial proteins and enzymes, alteration in DNA replications [131,132]; (iii) alteration of cellular respiratory chain or / and signal-transduction pathways [133,134].

In addition to their intrinsic antibacterial activity, AgNPs proved impressive synergistic effects in the case of combined treatment with different natural or synthetic compounds. The treatment with PVA-capped nanosilver and hydrogen peroxide determined rapid and synergistic bactericidal effects against both Gram-negative and Gram-positive strains [169]. Biasi-Garbin and coworkers reported that bioreduced AgNPs combined with eugenol had enhanced inhibitory activity against planktonic and biofilm-embedded drug-susceptible and drug-resistant *Streptococcus agalactiae* isolates [170]. In comparison with conventional PVP-capped AgNPs, curcumin-capped nanosilver showed enhanced bacterial inhibitory and killing activity. The presence of curcumin determined superior interactions with bacterial cells and higher release of silver ions, resulting in ROS-mediated cytotoxicity [171].

Enhanced inhibitory effects on *E. coli* were reported when using the treatment with AgNPs, *Centaurea damascena* essential oil and Gentamicin or Amoxicillin. In the case of *S. aureus*, the most prominent synergistic effect was reported when combining nanoparticles and essential oil with Imipenem [172]. Pronounced antibacterial effects on both strains were also reported when treated with Vancomycin-loaded AgNPs [173].

Similar synergistic effects were reported on *S. aureus* and *P. aeruginosa* when treated with triangular-shaped nanosilver and Ampicillin or Gentamicin [174], but also when treated with Metronidazole-capped spherical AgNPs [175]. Streptomycin-resistant *B. subtilis* exhibited important susceptibility on the antibiotic-conjugated AgNPs treatment [176], while Ciprofloxacin-conjugated nanoparticles caused significant bactericidal effects against *S. epidermidis* [177]. Biosynthesized nanosilver conjugated with Ampicillin or Vancomycin [178] and Tetracycline [179] determined improved antibacterial effects against *K. pneumoniae*, whereas 10 nm sized bioreduced AgNPs proved potentiated antibacterial effects on *S. mutans* treated with Gentamycin and Vancomycin [180].

The exposure of *E. coli* and *S. aureus* to the combined treatment with AgNPs and visible light irradiation resulted in enhanced synergistic antibacterial effects against both strains. However, the ROS-mediated cytotoxicity was more evident in the case of Gram-negative bacterium [181]. Light-irradiated enhanced bactericidal effects were also reported for *P. aeruginosa* treated with citrate-coated nanoparticles [182].

## 4. Silver Nanoparticles for Antiviral Applications

Given the complexity of pathophysiological interactions established between healthy cells and viruses, the development of specific and effective antiviral agents requires thorough and unceasing efforts [183,184]. The presence of living cells is mandatory for the replication of viruses, which invade and impair or even destroy host cells. Acute and chronic conditions occurred after viral contamination generally cause systemic infections and severe related complications. Few antivirals (generally, inhibitory protein-specific or enzyme-specific drugs and nucleoside or nucleotide analogs that interfere with viral replication cycle) [185] and vaccines (biological formulations containing viral vectors—attenuated or inactivated organisms, toxins or proteins, nucleic acids or genes that activate the innate immune system of the host) [186,187] are currently available to treat viral infections. As a result of nanosize-guided structural and molecular complex studies, nanosilver-based biomaterials proved impressive tools for the development of specific, selective and efficient antiviral therapies.

The intrinsic antiviral mechanism of silver nanoparticles is not completely known and understood, the studies requiring more complex structural, molecular and immunological research than in the case of antibacterial properties. In a similar way with their antibacterial activity, the antiviral effects induced by AgNPs rely on the specific affinity for essential biomolecules (viral proteins and glycoproteins, enzymes, lipids, nucleic acids) and Ag^+^-mediated biostatic events, such as obstruction of cellular attachment and invasion, the arrest of intracellular viral replication or propagation, hinder of extracellular virions production [188,189,190].

Ultrasound-assisted biosynthesized AgNPs (5–15 nm) exhibited virucide activity against influenza A virus (IAV) at noncytotoxic concentrations [191]. Previous data demonstrated the size-related antiviral action of nanosilver against IAV [192]. Significant antiviral effects were reported for nanoparticles functionalized with IAV inhibitory peptide ligand due to the potentiating effect of released silver ions on the peptide [193]. AgNPs decorated with Oseltamivir and Zanamivir (inhibitors of surface-expressed neuraminidase enzyme) showed synergistic antiviral effects against IAV, by preventing attachment to host cells and hindering viral activity by downregulation in ROS generation [194,195]. Moreover, nanosilver proved to represent a suitable adjuvant for the virus-inactivated vaccine, resulting in reduced lung inflammation and induced mucosal immunity [196].

It was also reported that AgNPs interfere with the host cell attachment of respiratory syncytial virus (RSV). Curcumin-modified nanoparticles (11.95 ± 0.23 nm) significantly inhibited the infectivity of RSV, by interacting with envelope glycoproteins and thus blocking virus internalization by human epithelial cells [197]. Recently, Morris et al. demonstrated that 10 nm PVP-coated nanosilver reduced RSV replication and proinflammatory cytokines production, both in epithelial cell lines and infected mouse lung tissue [198].

Fungal bioreduced AgNPs proved to inhibit cellular attachment and intracellular replication of type 1 herpes simplex virus (HSV-1), in a manner dependent on the particle size [199]. Noncovalent interactions between HSV-1 thymidine kinase ligands and nanoparticles biosynthesized with plant extracts were considered responsible for the antiviral activity of nanosilver [200]. AgNPs modified with tannic acid (33 nm) showed the ability to reduce the cellular infectivity with type 2 HSV (HSV-2), by directly blocking viral glycoproteins and interacting with viral DNA. The treatment with these nanoparticles also reduced local inflammation and potentiated virus-specific immune response in both primary and recurrent HSV-2 infection of mice [201,202]. Noncytotoxic concentrations of nanosilver produced by marine alga effectively reduced the cytopathic effect (an indication of host cell death after virus-related lysis or reproductive inability) in cells infected with HSV-1 and HSV-2 [203].

PVP-coated AgNPs (25 nm) with antitumor activity exhibited high cytotoxicity on cells infected with oncogenic γ-herpesviruses, such as Kaposi’s sarcoma-associated herpesvirus and Epstein–Barr virus. The nanoparticles interfered with viral replication (by inducing ROS generation and activating autophagy) and impaired associated virions [204]. El-Mohamady and coworkers reported that cytocompatible concentrations of spherical AgNPs (<30 nm) induced inhibitory effects on the replication of bovine herpesvirus-1 [205].

It was previously reported that AgNPs show antiviral action against cells infected with type 1 human immunodeficiency virus (HIV-1) [206], but are also able to prevent cell infection [207]. Low concentrations of silver nanorods conjugated with sodium 2-mercaptoethane sulfonate significantly interfered with HIV-1 replication [208]. AgNPs (10–28 nm) biosynthesized with *Rhizophora lamarckii* extract inhibited the activity of HIV-1 reverse transcriptase, an essential viral replication enzyme [209]. It was determined that positively charged nanosilver can form complexes with either HIV-1 protease (able to split viral polyproteins into mature and infectious particles) or specific peptides (macromolecules similar to HIV-1′s polyproteins). Due to competitive interactions, the early presence of AgNPs resulted in the most important decrease in viral replication [210].

Nanosilver-based formulations proved efficient therapeutic effects against several pathologies caused by clinically-relevant viruses, such as severe acute respiratory syndrome coronavirus 2 (SARS-CoV-2) [211,212], human papilloma virus (HPV) [213], rotavirus [214,215] and other enteric viruses [216,217,218]. It is worth mentioning that new and effective platforms containing AgNPs were evaluated for their biocidal activity against viral vectors, generally mosquito-borne pathogens including Zika virus [219,220], Dengue virus [221,222], West Nile virus [223,224] and Chikungunya virus [225,226].

## 5. Silver Nanoparticles for Cancer Therapy

Cancer represents a major concern in public health, being a group of aggressive and treatment-deficitary diseases that are incriminated in an alarming number of deaths at a global level [227,228]. In general, the conventional treatment of cancers consists of strategies with reduced selectivity and specificity, such as surgery, radiation therapy and chemotherapy, which lead to inefficient anticancer therapy [229]. With the aim to enhance the patients’ response to the considered anticancer treatment and to improve their general healthcare status, new nano-related strategies were proposed and assessed for cancer therapy [230,231].

Silver nanoparticles have a special role in modern anticancer therapy, being explored for detection and diagnosis of malignant tumors [232,233], controlled and externally triggered drug delivery systems [234,235]. In a similar way with the antimicrobial activity of AgNPs, their efficiency against cancer cells require the cellular uptake of nanosilver, which can be acquired by diffusion, phagocytosis, pinocytosis and receptor-mediated endocytosis [236,237]. The size, morphology and surface properties of AgNPs are favorable for internalization by cancer cells, which results in local release of silver ions and oxidative stress [238,239]. Such events further cause the death of cancer cells, either by (i) apoptosis, which occurs due to alteration of mitochondria and generation of imbalance between antiapoptotic proteins and proapoptotic kinases, and (ii) structural and functional impairment of cellular substructures, which occurs due to specific interactions with silver nanoparticles and ions [240,241].

Mitochondrial-dependent apoptosis of lung adenocarcinoma cells treated with nanosilver biosynthesized in the presence of cotton leaf extract was reported in a study performed by Kanipandian and coworkers. They also evidenced that negatively charged spherical nanoparticles, with 13–40 nm physical size, induced cell cycle arrest [242]. Besides oxidative stress, the treatment of lung cancer cells with AgNPs synthesized with *Anemarrhena asphodeloides* medicinal plant extract also resulted in decreased cellular migration [243]. The latest nanoparticles also proved anticancer efficiency against human colon and breast cancer cell lines. In addition, pulmonary cancer cells treated with biosynthesized AgNPs overexpressed the pro-apoptotic caspase-3 gene [244,245].

Under biological media, AgNPs may undergo specific processes that may influence their cytotoxicity, such as surface oxidation, biomolecule conjugation or attachment, the release of surface metallic ions [246,247]. In a complex comparative study performed by Ahn and coworkers, nanosilver obtained with thirty medicinal plant extracts exhibited substantial cytotoxicity against lung cancer cells, the results being remarkable in comparison with sole extracts. The authors also reported increased toxic effects in the case of cells cultured in media containing fetal bovine serum, as a consequence of protein corona modulated interactions [248]. Majeed et al., also reported that nanosilver resulting from bacteria-mediated biosynthesis and capped with bovine serum albumin showed important toxicity against breast cancer, colon carcinoma and osteosarcoma cells. In comparison with the initial nanosilver, protein-capped AgNPs (11.26–23.85 nm dimensional range) exhibited increased toxicity at reduced concentrations [249].

Well-dispersed AgNPs (20–30 nm size), obtained with tamarind fruit shell extract, induced apoptotic death in human breast cancer cells. A dose-dependent anticancer effect was reported, as the local increase of ROS led to mitochondrial impairment and DNA damage [250]. The same cytotoxic effects were evidenced after cellular treatment with AgNPs biosynthesized with extract from *Ochradenus arabicus* medicinal shrub [251] and marine bacilli [252]. Synergistic toxicity against breast cancer cells were reported by using Capecitabine-loaded citrate-capped AgNPs [253] and Gemcitabine-loaded PVP-stabilized nanosilver [235].

Concentration-dependent cytotoxicity of AgNPs (33 nm average size) biosynthesized with extract of *Nepeta deflersiana* medicinal plant against human cervical cancer cells was reported. In a similar way to previous studies, the AgNPs-mediated oxidative stress was responsible for mitochondrial damage and cell cycle impairment, which further caused the apoptotic and necrotic death of malignant cells [254]. Electrolytically deposited AgNPs capped with black tea extract also proved anticancer efficiency [213]. Sinigrin-mediated synthesized AgNPs, with 20 nm average size, induced dose-dependent toxicity on cervical cancer cells, as well as synergistic apoptotic processes in the case of combined treatment with Camptothecin [255].

Medicinal plant extracts contain substantial amounts of secondary metabolites with important effects against tumor cells. Highly stable spherical AgNPs obtained using neem leaf and shrub root extracts showed toxicity against breast, colon and hepatic cancer cells. Still, the most reliable results were obtained when using ethanolic extracts (instead of aqueous) on the colon adenocarcinoma cell line [256]. Complementary results on the anticancer efficiency were reported for nanosilver obtained with bulletwood fruits extract [257]. AgNPs biosynthesized by freshwater cyanobacterium potentiated the antibacterial effects of commercial antibiotics in the case of combined administration. In addition, they showed dose-dependent cytotoxic effects against human breast and colon cancer cells, apoptotic events being evidenced [258].

In another study, biogenic AgNPs prepared by using honey from distinctive floral sources manifested antiproliferative activity against liver tumor cells [259]. Quasispherical silver nanoparticles biosynthesized with lotus extract showed significant cytotoxic effects against human prostate, liver and gastric cancer cells [260]. Gastric adenocarcinoma cells were also impaired after treatment with AgNPs biosynthesized with medicinal extracts from leaves of felty germander [261] and from fruits of *Crataegus microphylla* shrub [262]. Other recent data on the toxic effects of silver nanoparticles against cancer cells are included in Table 3.

Besides their effects on cellular and subcellular structures, AgNPs significantly affect tumor angiogenesis, being responsible for alterations in growth factors’ expression and subsequent restricted proliferation and migration of endothelial cells [282,283]. Yang and coworkers reported that ~10 nm AgNPs induced dose-dependent apoptosis in breast cancer cells but also inhibited the transcription of hypoxia-inducible factor-1 (HIF-1) and the induction of vascular endothelial growth factor-A (VEGF). Together with the inhibition of tube formation in healthy endothelial cells, the authors proved the antiangiogenic effects of nanosilver [284]. Another study evidenced that the inoculation of AgNPs within the chorioallantoic membrane caused an important increase in the expression of caspase-3 and caspase-8 genes, which are responsible for cell apoptosis. The 15 nm particles obtained with red amaranth extract induced an important decrease in the length and number of new blood vessels and showed cytotoxic effects against breast malignant cells [285]. Also, antiangiogenic effects were reported for chicken chorioallantoic membrane treated with AgNPs biosynthesized with madder extract [286].

Except for their intrinsic anticancer effects, particular attention was oriented to the development and assessment of new silver-based nanosystems for boosted chemotherapy and radiotherapy. For example, branched gold-silver nanoparticles coated with dopamine and subjected to near-infrared irradiation determined photothermal-mediated cytotoxicity against colon cancer cell lines. By complex *in vitro* and *in vivo* studies, the authors evidenced that NP-mediated photothermal therapy (PTT) occurred by various apoptotic and necrotic mechanisms [287]. Multifunctional core-shell nanosystems based on AgNP core and aggregation-induced emission molecule were recently proposed by He and coworkers. The complex platforms were excellent enhancers for radiotherapy and modulators for PTT and photoacoustic effect but also exhibited excellent potential as contrast agents for fluorescence and computed tomography imaging [288].

Systems based on silver/magnetite nanoparticles coated with PEG, modified with folic acid and loaded with Doxorubicin drug showed great potential for the PTT of cervical cancer. Besides dual chemotherapeutic/photothermal effects, the hierarchical platforms exhibited targeted specificity for cancer cells and imaging potential by fluorescence and magnetic resonance [289]. Due to synergistic chemotherapeutic and photothermal effects that occurred after laser irradiation, enhanced cytotoxicity against malignant cells was reported for nanosystems based on Methotrexate-conjugated nanoparticles based on graphene oxide (GO) and AgNPs [290] and 5-Fluorouracil-loaded mesoporous SiO_2_-coated silver/gold nanoshells [291].

AgNPs capped with PEG and labeled with I-131 radionuclide showed increased targeting ability for malignant tissues in an animal model, with maximum solid tumor uptake of 35.43 ± 1.12% ID/g (reached at 60 min. after intravenous inoculation) and 63.8 ± 1.3% ID/g (reached at 15 min. after intratumor injection) [292]. Biosynthesized AgNPs with intrinsic cytotoxicity against hepatic malignant cells, proved a potentiating effect on the gamma radiation treatment [293]. Also, the treatment with cold atmospheric plasma-activated PVA-stabilized nanosilver resulted in additive cytotoxic effects against human glioblastoma multiforme cells [294].

AgNPs obtained in the presence of globe artichoke (*Cynara scolymus*) leaf extract by microwave irradiation showed the photosensitizing ability for the photodynamic therapy (PDT) of human breast adenocarcinoma cells. Synergistic effects were reported following the combined treatment, such as severe mitochondrial damage and ROS generation [295]. Enhanced cytotoxic effects were also reported in the case of melanoma cells, where AgNPs functionalized with porphyrin acted as mediators for enhanced PDT [296].

## 6. Silver Nanoparticles for Tissue Engineering

At the microstructural level, human tissues consist of highly organized cells with specific functions and their corresponding extracellular matrix (ECM, protein-based environment containing glycosaminoglycans, which in turn provides three-dimensional support for cellular adhesion and proliferation, regulates intercellular communication and tunes cell physiology). Generally, the structural and functional impairment of human tissue may occur due to acute or chronic injuries, severe inflammatory conditions, genetic disorders, degenerative conditions and tumors. With the aim to overcome the limitations of organ transplantation (including reduced bioavailability in the case of autografts and isografts, immunogenicity and graft rejection in the case of allografts and xenografts), healthcare professionals and scientists turned their attention towards the impressive potential of tissue engineering.

As a part of regenerative medicine, the desideratum of tissue engineering (TE) is represented by the fabrication of nonviable complex biocompatible systems that are able to revive the structural integrity and functionality of damaged tissues by restoring, replacing or regenerate them [297]. Nanostructured biomaterials represent a suitable choice for TE applications, not only because they properly interact with living systems and possess specific and selective therapeutic purpose, but also because they possess versatile and tunable characteristics which enable the achievement of particular requirements, such as (i) biocompatibility (a complex feature that relies on the bidirectional interactions between nanomaterials and host cells or tissues); (ii) physicochemical properties (microstructure, phase transitions, porosity, wettability, morphology, topography, composition, stability, reactivity); and (iii) circumstantial bioactivity [298,299].

Given their reduced toxic effects in healthy cells, facile surface functionalization and excellent antimicrobial activity, the impact of nanosilver-based biomaterials for TE was thoroughly evaluated. 

To begin with, AgNPs-embedded coatings were reported to boost the biological performances of bioinert materials used in orthopedics and orthodontics. The simple modification of titanium implants’ surface with nanosilver resulted in significant antibacterial effects against strains responsible for implant-associated infections while maintaining excellent biocompatibility [300,301,302]. Nanotubular titanium oxide surface coated with silver nanowires showed prolonged inhibitory action against *S. aureus* and *E. coli*, with more prominent effects against the Gram-positive strain. At the same time, the nanostructured surface exhibited protein adsorption capacity and proved an excellent substrate for the adhesion and proliferation of osteoblast-like cells [303]. Similar bactericidal performances were also evidenced for a mixed titanium/niobium oxide nanotube array coated with AgNPs-decorated GO sheets. In comparison with bare titanium-based alloy, the as-modified substrates showed improved cytocompatibility and differentiation of pre-osteoblastic cells, alongside superior corrosion resistance and apatite formation ability [304].

Polymer coatings embedded with AgNPs are unharmful materials for normal cells and only act as protective carriers or enhancers for local anti-infective effects, thus inducing or potentiating the antibacterial activity of metallic biomaterials [305,306]. Titanium implants modified with nanosilver-embedded poly(lactic-*co*-glycolic) acid (PLGA) coatings showed strong bactericidal activity against opportunistic pathogens, together with important osteoinductive potential [307]. Nanocomposite coatings of chitosan–tragacanth gum embedded with nanoparticles of silica and biosynthesized silver (SiO_2_@Ag) demonstrated enhanced apatite-forming ability, as well as good antibacterial effects under both acidic and aqueous media [308]. Electrospun composites based on polylactic acid (PLA), GO and AgNPs increased the mechanical properties and anticorrosive behavior of magnesium alloy and encouraged the formation of a stable apatite layer. Such composite coatings reduced the degradation rate of magnesium alloy and proved beneficial for the adhesion, proliferation and normal development of osteoblast-like cells while significantly inhibited bacterial growth [309].

A more attractive and successful strategy to enhance the performance of metallic implants consists of modifying their surfaces with biomimicking coatings containing nanosilver, which simultaneously determine superior osseointegration and anti-infective efficiency [310,311]. Estrada-Cabrera and coworkers reported the potential use of composite coatings based on bioactive glass, CS and AgNPs for surface modification of anodized titanium implants [312]. Nanostructured material composed of hydroxyapatite (HAp), CS, AgNPs and lysozyme proved cytocompatible substrates for osteoblasts. Titanium substrates modified with such hybrid coatings exhibited strong bactericidal effects due to the synergistic activity of the latter two components [313]. Even if nanostructured coatings of HAp, zirconium oxide and nanosilver proved to decrease the corrosion resistance of zirconium/titanium alloy, they showed superior osteoconductive ability and enhanced the in vivo osseointegration of as-modified implants [314,315].

Significant inhibitory activity against planktonic and sessile bacteria was reported in the case of AgNPs-incorporated silk fibroin (SF) films. Low concentrations of nanosilver determined favorable cytocompatibility on fibroblasts and osteoblasts, as well as nondetrimental effects on the osteogenic differentiation ability of human mesenchymal stem cells [316]. The bone-forming potential of SF coatings embedded with AgNPs and Gentamycin was evidenced in an animal model. The highly hydrophilic and protein adsorptive surfaces showed a pH-dependent release of metallic silver, which determined enhanced biocompatibility, mineralization and osteoinductive potential, but also long-term antibacterial efficiency [317]. The potential use of hydrogels of SF and CMC loaded with low concentrations of nanosilver for TE applications was also reported. The highly absorbent composites showed strong antibacterial and mild antifungal efficiency and displayed cytocompatibility with respect to bone marrow stem cells [318].

A recently developed endodontic sealer based on methacrylate derivative embedded with nanoparticles of amorphous calcium phosphate and silver determined remineralization and strengthening effects on dentin, but also strong bactericidal activity against pathogens associated with dentin infections [319]. AgNPs-loaded natural rubber latex showed low toxicity and tissue reaction similar to commercial products, being proposed as an antibacterial occlusive membrane for guided bone regeneration in orthodontics [320]. High bactericidal efficiency and cytocompatibility were also reported by embedding rigid poly(methyl methacrylate) (PMMA) nanoparticles decorated with CS-stabilized AgNPs within soft films of natural rubber [321].

Biodegradable electrospun membranes of poly(caprolactone) (PCL) or polylactide/cellulose acetate (PLA/CA) embedded with nano-HAp and AgNPs promoted the formation of bone-like apatite. The nanofibrous composites exhibited prolonged bactericidal effects, being proposed as suitable materials for orthodontic applications [322]. Liu et al., reported the successful fabrication of nanofibrous materials based on PLA/HAp nanowires modified with polydopamine membrane and coated with polypyrrole-stabilized AgNPs. The obtained hybrid biomaterials possessed good stability under physiological conditions, enhanced mineralization ability, excellent cytocompatibility and long-term antibacterial efficiency, being promising candidates for bone-related regenerative and anti-infective applications [323].

The sustained release of Ag^+^ from PLA scaffolds modified with nanosilver-loaded halloysite nanotubes determined prolonged antibacterial activity. The as-modified scaffolds showed increased mechanical properties, degradability and mineralization, which positively contributed to supporting cellular proliferation and osteogenic differentiation [324]. Hasan and coworkers reported that CS/CMC scaffolds modified with AgNPs-decorated cellulose nanowhiskers possess suitable porosity and compressive behavior for bone TE applications, in conjunction with intrinsic antibacterial efficiency. The controlled degradability of hybrid scaffolds was adjusted for angiogenesis and vascularization processes and proved beneficial for *in vitro* mineralization, while the protein adsorption ability determined superior adhesion and proliferation of osteoblasts [325]. Silver nanorods incorporated within highly porous wollastonite scaffolds determined strong bactericidal effects while providing the favorable apatite-forming ability and good cytocompatibility with respect to osteoblast-like cells [326]. Composite freeze-thawed gelatin/alginate/PVA and electrospun PCL scaffolds embedded with a bactericidal concentration of silver-HAp nanoparticles showed suitable porosity and prolonged release metallic ions, with simultaneous favorable adhesion, proliferation and osteogenic potential on mammalian cells [327,328].

Substrate roughness and wettability possess a very important role in protein absorption and cellular attachment, therefore significantly contributing to the biological performance of implanted devices. The addition of AgNPs within electrospun scaffolds of CMC/PVA and PCL loaded with rambutan polyphenolic extract determined higher cellular proliferation rates due to surface modification [329,330]. Titanium oxide nanotube array coated with AgNPs-embedded polydopamine layer was assessed as a feasible option for arthroplasty [331]. CS-SF/PET (polyethylene terephthalate) scaffolds modified with nanosilver/HAp by plasma splashing procedure promoted cellular proliferation and osteogenic differentiation of mesenchymal stem cells. The composite scaffolds restricted the resorption of bone passage and enhanced the biomechanical response of bone–joint interface, being potential candidates for the replacement of anterior cruciate ligament [332]. In addition, AgNPs-decorated nanofibrous membranes of PET, with good cytocompatibility and inhibitory effects against planktonic and sessile bacteria, induced weak inflammation and reduced foreign body response in an animal model [333]. Poly(acrylonitrile-butadiene-styrene) copolymers modified with AgNPs were proposed as suitable candidates for the fabrication of middle ear implants. The composites exhibited pronounced hydrophilicity and long-term mechanical stability while determined no cytotoxic effects and promoted cellular proliferation in both osteoblast and fibroblast cultures [334,335].

Thanks to their excellent mechanical strength, gradual degradation and biological activity, agarose scaffolds impregnated with CS-coated nanosilver were proposed for soft TE applications. In addition to their intrinsic bactericidal efficiency, the biopolymer-based scaffolds showed good hemocompatibility and enhanced cytocompatibility with different epithelial cell lines [336]. The incorporation of AgNPs within composite aerogels of bacterial cellulose (BC) and polyaniline (PANI) determined increased viscoelastic behavior, which is an important factor for the repair and regeneration of soft tissue [337,338]. With the aim to obtain low-cost antibacterial scaffolds for TE applications, the decellularized fish swim bladder matrix was modified with colloidal AgNPs. The resulted collagen-enriched scaffold showed broad-spectrum bactericidal efficiency (due to the gradual release of nanosilver) and biocompatibility, as well as good flexibility and biodegradability [339]. The ultrastructure of decellularized esophageal scaffolds (more regular and enhanced binding of collagen fibers, reduced alteration of pore areas) was improved by modification with 5 µg/mL of citrate-stabilized AgNPs (100 nm), due to their ability to non-covalently interact with the collagenous material. The as-modified scaffolds presented superior water uptake ability, substantial resistance to enzymatic degradation and thermal stability, together with excellent anti calcification effect. Moreover, the nanosilver-modified matrices exhibited highly compatible behavior with respect to stem and endothelial cells, while the intrinsic anti-inflammatory activity of AgNPs led to a reduced immune response of tissue after *in vivo* implantation [340].

The interconnected porosity of CS scaffolds incorporated with AgNPs-embedded CS microspheres proved beneficial for the adhesion, proliferation and migration of fibroblasts. Given the sustained release of metallic silver and the prolonged antibacterial effects of such nanostructured scaffolds, they were evaluated as suitable candidates for skin TE by Niu and coworkers [341]. Macroporous CS sponge embedded with polysaccharide biosynthesized nanosilver were proposed for the regeneration of skin defects due to excellent water retention property, mechanical behavior and biological performances [342]. SF nanofibrous mats modified with biosynthesized AgNPs were also proposed for skin TE. In comparison with several commercial products, the highly biocompatible constructs showed superior extensibility and flexibility, as well as increased water uptake, which are essential aspects for tissue repair [343].

Thanks to their impressive compositional versatility and high intrinsic hydrophilicity, swelling capacity and tunable degradability, adequate elasticity and flexibility and stimuli-responsive ability, hydrogels attracted particular attention in regenerative medicine. AgNPs-embedded biocompatible platforms with promising potential for TE applications include guar gum hydrogel [344], gelatin/PEG/dopamine hydrogel [345] and carboxymethyl starch/PVA/citric acid hydrogel [346].

## 7. Silver Nanoparticles for Wound Care

Wounds are defined as damage in the natural structure of the skin and adjacent tissues, which may appear through several traumas, including physical or mechanical injury, chemical or thermal damage and biological impairment. The natural healing process starts right after the occurrence of a wound, by impressive local recruitment of immune, cellular and vascular components that synergistically act for the proper restoration of structural and physiological functions [347,348]. This process relies on the accurate sequence of the following essential stages: hemostasis, inflammation, cellular proliferation, re-epithelialization and tissue remodeling [349,350]. When the affected tissue is not able to heal properly, the wound healing process is inadequate and may further lead to various complications and even life-threatening conditions.

Currently, few strategies are available for the clinical management of wounds. For instance, in terms of compatibility and enhanced healing process, skin autografts and xenografts represent a desirable therapeutic choice for severe wounds. Except for being expensive approaches, these strategies have specific limitations, such as restricted bioavailability, respectively immunogenicity and increased possibility for disease transmission [351,352]. In addition, oxygen-enriched therapy is beneficial for accelerated wound healing, as oxygen is essential for the stimulation of collagen synthesis and subsequent re-epithelialization, as well as for the induction of angiogenesis [353,354]. Besides being a costly and uncomfortable procedure, it was also reported that hyperbaric oxygen therapy has limited efficiency since negative pressure therapy is generally suitable for small wounds and may induce several physical effects that can hamper the healing process [355,356]. Another therapeutic strategy for wound healing consists in using wound dressings, which support the structural and functional restoration of the injured tissue and may additionally provide protection against external pathogens. Several key aspects must be considered for an effective wound dressing, such as biocompatibility, fluid (super)absorption, water and oxygen partial permeability, nonimmunogenicity, facile and nontraumatic removal [357,358]. Even if a wide variety of dressings is commercially available, the current tendency in wound care management is to develop specific and performance-enhanced dressings, which provide suitable compositional, structural and biofunctional features for proper wound healing process [134,359].

The opportunistic microbial contamination and colonization of wounds generally lead to the delayed and circumstantial improper healing process, but it may also lead to severe infections and critical healthcare complications [360,361]. Therefore, impressive attention was oriented on the development of anti-infective wound dressings, which can be produced by embedding dressing materials with different antimicrobial agents, such as synthetic antibiotics [362,363], essential oils [364,365] and antibacterial nanoparticles [366,367,368].

Silver derivatives were used for wound care since ancient times, as even Hippocrates described their efficiency in wound healing [369]. Also, silver-based compounds were used to reduce the intraoperative risk of wound infection from the late XIX century [370] and remained the preferred agent for partial burns treatment since mid of XX century (as they can absorb fluids better and reduce infective processes) [371,372]. However, the decrease observed in the use of SSD-based products (occurred due to different side effects, like eschar formation and tissue irritation) led to the development of unconventional silver-based therapeutic formulations [373,374]. Among noble metals, silver, in the form of nanoparticles and nanosystems, represents the most explored representative for the successful development of innovative and effective wound dressings, thanks to their impressive biocide and biostatic effects, anti-inflammatory activity and reduced or absent toxicity for human tissues [37,375]. Given the above-mentioned aspects, together with tunable surface chemistry, drug delivery ability and low production costs [376,377], different products containing silver nanoparticles and ions are commercially available (as previously summarized in Table 1). Other silver-based nanostructured candidates with promising preclinical performances in wound care management are included in Table 4.

Depending on the type and localization of tissue injury, cotton dressings, silk sutures and synthetic polymeric mats represent the preferred choice for open wounds. Still, these formulations are effective only for promoting or accelerating the healing process, without preventing or eliminating opportunistic microbial contamination. The facile modification of cotton [378,379] and silk [380,381] materials with nanosilver resulted in promising local antibacterial effects without affecting the intrinsic long-term stability and wound healing ability of initial substrates. Cotton fabrics coated with AgNPs stabilized with CS derivatives exhibited strong and long-lasting bactericidal efficiency [382,383]. Substantial antimicrobial activity was also reported in the case of nanosilver-decorated polypropylene [384,385] and nylon [386,387] fibers and fibrous mats.

As a consequence of synergistic anti-infective efficiency, strong antimicrobial and anti-biofilm effects were reported for textile wound dressings modified with alginate embedded with AgNPs conjugated with essential oils of mandarin, clove and niaouli [388]. Wound dressings coated with biosynthesized AgNPs determined intense collagen deposition and faster re-epithelialization in the case of burn wounds (17 days, in comparison with 25 days for uncoated dressings). The as-modified materials exhibited improved tensile strength and accelerated healing potential, being thus proposed for the management of pediatric wounds [389].

The mechanical properties and antibacterial activity of CS films were experimentally improved by incorporating them with biosynthesized nanosilver [390,391]. CS films embedded with AgNPs were proposed as temporary wound dressings. The bacteriostatic and bactericidal composites showed a water vapor transmission rate comparable with commercial dressings, as well as reduced degradability and prolonged cytocompatibility on human fibroblasts [392]. Moreover, CS derivative films incorporated with AgNPs in 0.125% inorganic/organic ratio showed suitable hemolytic and hemostatic effects for wound healing applications [393].

The capacity of composite films of sodium alginate containing SiO_2_-coated AgNPs was investigated for wound dressing use. The obtained films showed slow release of silver ions, increased hydration properties and prolonged bactericidal and anti-biofilm activity. Even if comparable antibacterial effects were reported in the case of films incorporating acetate-stabilized nanoparticles, only the presence of silica layer determined excellent compatibility with respect to fibroblasts and keratinocytes [394]. The ability to promote wound healing and exert efficient antibacterial effects against planktonic and biofilm-embedded bacteria were also reported for alginate/HA membranes embedded with Chitlac-stabilized AgNPs [395].

Nanofibrous electrospun mats of hyaluronic acid (HA) and polygalacturonic acid (PGA) embedded with nanosilver showed promising results for wound healing. Increased mechanical behavior and hydrophilicity, but also enhanced antibacterial activity was reported for the composites obtained by electrospinning. Moreover, faster healing and wound epithelialization were evidenced, as well as reduced tissue inflammation. Such behavior was assigned to the presence of AgNPs, which intrinsic anti-inflammatory activity contributed to accelerated wound healing [396].

The hydrophilic nature of hydrogels is closely related to their impressive ability to absorb wound exudates and to maintain adequate wound bed moisture [397,398]. By also considering their intrinsic flexibility and swelling, similar hydration with skin tissue and circumstantial stimuli-responsive ability, (hydro)gel dressings help to substantially reduce pain scores, accelerate wound healing and prevent bacterial contamination by the facile incorporation of antimicrobial agents [358,399].

In order to extend the use of CS hydrogels for wound dressing applications, Wang and coworkers modified them with AgNPs. They demonstrated that the addition of nanoparticles within biopolymeric hydrogel resulted in ultrahigh mechanical properties of the composite hydrogel without affecting its structural integrity. Faster and improved wound healing were evidenced in the case of nanosilver-embedded hydrogel [400]. Also, biocompatible PVA/CS hydrogels loaded with electrochemically synthesized nanosilver showed improved swelling ability, as well as the slower and prolonged release of metallic ions, which are desirable aspects for wound healing applications [401]. Together with favorable mechanical strength and self-healing ability, the excellent biocompatibility and enhanced healing effects evidenced on infected wounds recommend AgNPs-loaded chitosan/carboxymethyl chitosan hydrogel for wound management [402].

Composite hydrogels of alginate/gelatin loaded with AgNPs determined the improved formation and maturation of granular tissue and promoted the earlier formation of primary collagen scars [403]. Effective antibacterial activity accelerated healing process and enhanced re-epithelialization were reported in the case of PVA hydrogel patches loaded with biosynthesized AgNPs [404]. Thanks to their enhanced water vapor transport and increased moisture retention, highly antibacterial starch/PVA hydrogel membranes loaded with biosynthesized AgNPs exhibited impressive potential for wound dressing use [405]. Jaiswal and coworkers demonstrated the promising use of carrageenan hydrogel films embedded with lignin-stabilized nanoparticles for the treatment of full-thickness wounds [406]. The efficiency of Pluronic F-127 gels loaded with nanosilver against planktonic and sessile bacteria was also reported. The proposed formulations showed great cytocompatibility on human cells and excellent thermoreversibility, which determined the facile application of gel dressing [407].

In a complex study, Erring et al. investigated the healing ability of AgNPs gel and nanosilver foam dressings in patients with burn wounds. In comparison with nanostructured gel and collagen dressings, the nanostructured foam exhibited the best results: faster-wound healing rate, a higher number of patients with improved re-epithelialization (55% vs. 20% and 30%, respectively), increased ease of application (95% vs. 78% and 80%, respectively), improved level of tolerance and significantly reduced pain scores. All taken together, nanosilver foam dressings proved a more efficient strategy for the management of partial-thickness burns [18].

Besides their complex cellular compatibility (which enables adhesion, proliferation and circumstantial differentiation) and porous architecture (which is beneficial for cellular migration and angiogenesis), 3D scaffolds designed for wound healing applications possess proper mechanical features, suitable flexibility, swelling ability and tunable biodegradability [134,408].

Highly organized collagen scaffolds stabilized with juglone-functionalized AgNPs were evaluated as beneficial substrates for cellular adhesion and intercellular connection. The as-obtained nanostructured scaffolds exhibited significant antiproteolytic and proangiogenic ability and determined faster and enhanced wound healing [409]. Bergonzi et al. recently proposed 3D-printed scaffolds of alginate and nanocrystalline cellulose incorporated with nanosilver as highly absorbable and elastic macroporous scaffolds with considerable antibacterial effects for wound care applications [410]. AgNPs-impregnated BC/polydopamine scaffolds were proposed for the management of burn wounds, with complete healing being evidenced after 25 days. The nanocomposites facilitated necrotic tissue clearance, promoted collagen deposition and epidermis neoformation. The scaffolds also determined increased/decreased levels of anti-inflammatory/pro-inflammatory interleukins, respectively, and upregulation of growth factor genes involved in wound healing [411]. Highly antimicrobial electrospun PLA scaffolds modified with nanosilver and cellulose nanofibrils promoted the proliferation and normal growth of ocular epithelial cells, with no proinflammatory reaction. The hydrophilic scaffolds were recently proposed as effective ocular bandages [11]. AgNPs and lavender oil-induced synergistic antibacterial effects when incorporated within polyurethane (PU) nanofibrous scaffolds. The resulted hydrophilic nanocomposites encouraged improved proliferation and normal development of fibroblasts [412].

Diabetic wounds are moderate to severe chronic wounds which natural healing is generally disturbed by several disease-associated factors, such as glycemic levels and local hypoxia, peripheral vascular disease and neuropathy, compromised immunodeficiency and opportunistic infections [424]. The successful use of silver-based nanomaterials in wound therapy relies on their ability to be specifically modified and easily incorporated within dressing materials (which facilitates transportation, protection and control of therapeutic agents), respectively, on the improvement of skin remodeling through antioxidant, anti-inflammatory and proliferative properties [425,426]. Nanosilver has the advantage of better chemical stability and catalytic activity, thus being used as an intrinsic therapeutic agent. The limitation of using AgNPs in wound healing consists of their toxicity rate, but this aspect can be tuned by raising the surface-to-volume ratio and by different protective coatings [427]. Taking all into consideration, particular attention was straightened to the use of AgNPs in the management of diabetic wounds.

The mid-term use (three days) of AgNPs-incorporated chitin nanofiber sheet after wound bed cleansing with weakly acidic hypochlorous acid (pH 6.5) determined enhanced disinfection and significant healing efficiency in mice diabetic wounds infected with *P. aeruginosa* [428]. Hybrid hydrogels of thiolated CS and dextran grafted with maleic acid embedded with AgNPs were proposed as effective dressings for diabetic ulcers. The proposed antifouling hydrogel showed macroporous architecture and excellent water absorption ability while promoted and accelerated the healing process and modulated the host immune response by the local recruitment and activation of immune cells [429]. The slow and sustained release of metallic particles from CS/PEG hydrogels loaded with AgNPs proved beneficial implications on the prolonged antibacterial efficiency and accelerated healing of wounds in diabetic rabbits. The highly porous nature and increased swelling ability of composite hydrogel contributed to faster complete re-epithelialization (12.3 ± 0.8 days) and collagen deposition processes (34.4 ± 2.0 on day 12), while resulted in moderate granulation tissue and reduced inflammation. After 14 days of experimental treatment, blood vessels and nuclei formation were also noticed, an indication the angiogenic potential of nanosilver-embedded CS/PEG hydrogels [430].

## 8. Concluding Remarks

The genuine size-related physicochemical features, mechanical and optical properties and peculiar biological behavior (including nontoxicity, antimicrobial efficiency and biofunctional ability) represent fundamental characteristics that recommend silver nanoparticles (AgNPs) for the development of unconventional and effective biomedical applications. For such particular use, a real challenge for researchers is to properly tune the biocompatibility/antimicrobial activity balance as to maximize the desired therapeutic effects. Thanks to their impressive versatility, nanosilver-based biomaterials and biosystems are promising candidates for unconventional anti-infective therapy, specific and selective platforms for detection and diagnosis, targeted and controlled drug delivery and gene therapy, soft and tissue engineering and regenerative medicine.

## Figures and Tables

**Table 1:** Commercial products containing ionic (Ag^+^) or metallic silver (Ag^0^/AgNPs).

Product Type	Product Trademark	Company	Note
Wound dressing	Acticoat™	Smith & Nephew, Inc. (London, UK)	Flexible and nonadherent nanocrystalline silver dressing Provides sustained broad-spectrum bactericidal activity against over 150 strains
	Allevyn™ Ag		Absorbent and flexible silicone foam dressing impregnated with SSD Provides sustained long-term bactericidal effects
	Actisorb™ Silver	3M+KCI (MN, USA)	Activated charcoal layer impregnated with silver Provides anti-bacterial barrier action and bactericidal activity
	Silvercel™		Nonwoven pad of natural polysaccharides blend and nylon fibers impregnated with ionic silver Provides sustained long-term bactericidal and anti-biofilm effects
	Tegaderm™ Alginate Ag		Absorbent soft-gelling alginate dressing impregnated with silver Provides sustained long-term broad-spectrum bactericidal effects
	Maxorb^®^ Extra Ag^+^	Medline Industries, Inc. (IL, USA)	Blend fibers of natural polysaccharides impregnated with ionic silver Provides a sustained and long-term barrier against bacteria absorbed in wound exudates
	Opticell^®^ Ag^+^		Absorbent and flexible gelling fiber format impregnated with ionic silver Provides sustained long-term bactericidal activity
	SilvaSorb™ Sheet		Super-absorbent hydrogel sheet impregnated with ionic silver Provides sustained long-term bactericidal activity
	SilvaSorb™ Gel		Hydrogel ointment impregnated with ionic silver Provides sustained broad-spectrum antimicrobial action
	Aquacell^®^ Ag	ConvaTec Group (Deeside, UK)	Nonwoven inner pad impregnated with ionic silver Provides long-term broad-spectrum bactericidal and bacteriostatic effects
	PolyMem Silver™	Ferris Mfg. Corp. (TX, USA)	Foam dressing impregnated with nanocrystalline silver Provides fungicidal and broad-spectrum bactericidal effects
	SilvrSTAT^®^	ABL Medical (UT, USA)	Hydrogel dressing ointment impregnated with metallic silver Provides antimicrobial action in first- and second-degree burns
Catheter coating	Silverline^®^ Drainage Catheters	Spiegelberg GmbH & Co. (Hamburg, Germany)	Radiopaque polyurethane or silicone catheters modified with silver Provides antimicrobial and anti-biofilm effects in the case of drainage in central nervous system structures
	Covidien^®^ Foley Catheter	Medtronic (London, UK)	Outer and inner silicone catheter and balloon coated with ionic silver hydrogel coating Provides substantial antimicrobial activity by consistent release of ionic silver
	SilverSoaker™ Catheter	Halyard Health, Inc., (GA, USA)	Outer and inner catheter coated with metallic silver (SilvaGard™) Provides antimicrobial and anti-biofilm effects
	Bardex^®^ Catheter	C.R. Bard Inc., (NJ, USA)	Latex Foley catheter modified with Bard^®^ hydrogel and Bactiguard^®^ silver coating Provides antibacterial and anti-biofilm effects
Endotracheal tube	Agento^®^ Silver-coated Endotracheal Tube	C.R. Bard Inc., (NJ, USA)	Endotracheal tube modified with a hydrophilic polymer coating containing silver particles Provides microbiological efficiency against ventilator-associated pneumonia

Abbreviation: SSD—silver sulfadiazine.

**Table 2:** Effects of AgNPs against various bacterial pathogens.

Bacterial Strain	Proposed Systems	Effects	Refs.
*Bacillus subtilis* (*B. subtilis*)	AgNPs biosynthesized with petai (*Parkia speciosa*), fig tree (*Ficus hispida*), pomegranate (*Punica granatum*), *Sida cordifolia* and *Platycodon grandiflorum* extracts	Antibacterial effect due to size-related cytotoxicity and phytochemicals	[135,136,137,138,139]
	AgNPs biosynthesized with coriander (*Coriandrum sativum*) leaf extract and AgNPs bioreduced by *Actinomycetes* strain	Bacterial death due to cellular uptake and Ag^+^-mediated DNA damage	[140,141]
*Enterococcus faecalis* (*E. faecalis*)	AgNPs biosynthesized with night-blooming jasmine (*Cestrum nocturnum*) extract	Bacteriostatic and bactericidal effects exhibited for lower and higher AgNPs concentrations, respectively	[142]
	AgNPs bioreduced by *Fusarium semitectum* strain	Strong antibacterial and anti-biofilm activity	[143,144]
*Klebsiella pneumoniae* (*K. pneumoniae*)	AgNPs biosynthesized with butterfly pea (*Clitoria ternatea*) and mango (*Mangifera indica*) flower extracts and wild ginger (*Alpinia nigra*) fruit extract	Antibacterial effect due to size-related cytotoxicity and phytochemicals	[145,146,147]
	AgNPs bioreduced by *Nostoc Bahar M.* cyanobacteria	Strong bactericidal effect due to imbalance in bacterial antioxidants and enzymes, fragmentation and degradation of bacterial proteins	[148]
	AgNPs bioreduced by *Bifidobacterium bifidum* strain	Antibacterial activity due to inhibitory effects on efflux pump genes	[149]
	PVP-capped AgNPs	Antibacterial effects due to membrane disruption and cytoplasmic protein leakage, anti-biofilm effects due to inhibitory activity on extracellular protein substances	[150]
*Pseudomonas aeruginosa* (*P. aeruginosa*)	AgNPs biosynthesized with sesame (*Sesamum indicum*) oil, horse chestnut (*Aesculus hippocastanum*) and stonebreaker (*Phyllanthus niruri*) extracts	Bacterial death due to cellular uptake and size-related intracellular toxicity	[151,152,153]
	AgNPs dendronized with cationic carbosilane dendrons and modified with PEG	Destabilization of outer membrane, degradation of peptidoglycan layer (in conjunction with endolysin)	[154]
	AgNPs biosynthesized with eyebright (*Euphrasia officinalis*) leaf extract	Strong antibacterial and anti-biofilm activity	[155]
	AgNPs biosynthesized with *Lysiloma acapulcensis* extract	Antibacterial effect due to size-related cytotoxicity and phytochemicals	[156]
*Salmonella enterica* (*S. enterica*)	AgNPs biosynthesized with green tea (*Camellia sinensis*) and jackfruit (*Artocarpus heterophyllus*) extracts	Synergistic inhibitory and bactericidal effects due to size-related toxicity and phytochemicals	[157,158]
	AgNPs capped with afzelin and quercitrin extracted from *Crotolaria tetragona*	Bacteriostatic and bactericidal effects, anti-biofilm activity due to alteration of membrane potential and efflux pumps and modification of bacterial surface hydrophobicity	[159]
	AgNPs bioreduced by *Penicillium polonicum* strain	Strong bactericidal activity due to membrane disruption and cytoplasmic protein leakage	[160]
*Staphylococcus epidermidis* (*S. epidermidis*)	AgNPs biosynthesized with river bushwillow (*Combretum erythrophyllum*) leaf extract, grape (*Vitis vinifera*) fruit extract and *Elytraria acaulis* leaf extract	Bacterial death due to cellular uptake and size-related intracellular toxicity	[161,162,163]
	AgNPs biosynthesized with tea tree (*Melaleuca alternifolia*) essential oil	Inhibitory and bactericidal effects due to membrane disruption and bacterial internalization, synergistic toxicity related to AgNPs size and tea tree essential oil	[164]
*Streptococcus mutans* (*S. mutans*)	AgNPs biosynthesized with citrus (*Citrus limetta*) peel extract	Antibacterial effect due to size-related membrane permeability alteration and anti-biofilm activity	[165]
	SiO_2_-coated AgNPs biosynthesized with green tea (*Camellia sinensis*) extract	Strong antibacterial and anti-biofilm activity	[166]
*Streptococcus pyogenes* (*S. pyogenes*)	AgNPs biosynthesized with *Dodonaea viscosa* extract and AgNPs bioreduced by *Saccharopolyspora hirsute* strain	Antibacterial effect due to size-related cytotoxicity and phytochemicals	[167,168]

**Table 3:** Cytotoxicity of AgNPs against various cancers.

Malignant Cells	Proposed Systems	Effects	Refs.
Bladder carcinoma	AgNPs bioreduced by *Fusarium oxysporum* strain	Apoptosis induced by DNA damage, reduced cellular migration and proliferation, tumor regression	[263]
Breast adenocarcinoma	AgNPs bioreduced by *Penicillium citrinum* strain	Apoptosis induced by DNA damage	[264,265]
	AgNPs biosynthesized with fineleaf fumitory (*Fumaria parviflora*), rhododendron (*Rhododendron ponticum*), rhubarb (*Rheum ribes*) and cumin (*Cuminum cyminum*) extracts	Cell death evidenced on distinctive tumor cell lines	[266,267,268,269]
Colorectal cancer	AgNPs biosynthesized with creeping woodsorrel (*Oxalis corniculata*) leaf extract	Cell death induced by apoptotic and necrotic mechanisms	[270]
	AgNPs biosynthesized with peacock (*Caesalpinia pulcherrima*) flower extract	Cell death induced by apoptosis and membrane damage	[271]
Hepatocellular carcinoma	AgNPs bioreduced by *Bacillus safensis* strain	Cell death induced by apoptotic and necrotic mechanisms	[272]
	PVP-stabilized AgNPs	Cell death induced by damage of cellular organelles (especially mitochondria) and oxidative stress, upregulation of mitochondrial proapoptotic proteins	[273]
Laryngeal carcinoma	AgNPs bioreduced by *Penicillium italicum* strain	Cell death induced by ROS-mediated membrane damage and essential enzymes impairment	[274]
Lung adenocarcinoma	AgNPs bioreduced by *Bacillus amyloliquefaciens* strain	Cell death induced by ROS generation and damage of cellular organelles	[275]
	AgNPs biosynthesized with soursop (*Annona muricate*) and mangrove (*Avicennia marina*) leaf extracts	Apoptosis induced by ROS generation, downregulation of antiapoptotic genes and upregulation of proapoptotic genes	[276,277]
Osteosarcoma	AgNPs biosynthesized with cempedak (*Artocarpus integer*) and mangrove (*Rhizophora apiculata*) leaf extracts and noni (*Morinda citrifolia*) bark extract	Cell death evidenced on distinctive tumor cell lines, cell death induced by membrane damage and oxidative stress	[278,279,280]
Rhabdomyosarcoma	AgNPs bioreduced by *Bacillus* sp. strain	Cell death induced by ROS generation	[281]

**Table 4:** Nanosilver-embedded formulations for wound healing.

Proposed Systems	In Vitro Effects	In Vivo Effects	Refs.
CS films embedded with CS-stabilized AgNPs	Antibacterial effects against *E. coli*	Better and faster wound healing rate, reduced local inflammation and enhanced angiogenesis	[413]
CS/sericin films conjugated with AgNPs and loaded with Moxifloxacin	Antibacterial effects against *E. coli*, *P. aeruginosa*, *S. epidermidis*, drug-sensitive and drug-resistant *S. aureus*	Rapid and enhanced repair of infected burn wounds accelerated wound healing, reduced local inflammation, improved collagen deposition and angiogenesis	[414]
CS/PEO nanofibrous membranes incorporated with AgNPs	Antibacterial effects against *S. aureus*	Bactericidal effects in infected wounds, faster wound healing rate, improved regeneration of epidermis and neovascularization	[415]
CS/KGM hydrogel embedded with AgNPs	Antibacterial effects against *E. coli* and *S. aureus* Good biocompatibility on fibroblasts	Enhanced repair of infected wounds, reduced inflammation by regulating local levels of proinflammatory and anti-inflammatory interleukins	[416]
Collagen/CS dressing loaded with AgNPs	Antibacterial effects against *E. coli*, *P. aeruginosa* and *S. aureus*	Faster wound healing rate, enhanced re-epithelialization, reduced local inflammation, downregulation of inflammatory cytokine and upregulation of growth factors	[417]
Galactoxyloglucan hydrogel scaffolds decorated with AgNPs	Antimicrobial effects against *E. coli*, *S. aureus* and *C. albicans* Enhanced cellular adhesion and proliferation of fibroblasts	Bactericidal effects in infected wounds, better and faster wound healing rate, improved collagen deposition and angiogenesis	[418]
PVA/β-cyclodextrin nanofibrous scaffolds loaded with AgNPs and riboflavin	Antibacterial effects against *E. coli* and *S. aureus*Enhanced cellular proliferation of epithelial cells	Enhanced wound contraction and re-epithelialization	[419]
PVA/PVP/pectin/MF nanofibers embedded with AgNPs	Antibacterial effects against *E. coli*, *P. aeruginosa* and *S. aureus* Enhanced cellular proliferation of fibroblasts	Faster healing rate and tissue regeneration	[420]
PCL/PVA nanofibrous scaffolds loaded with AgNPs	Antibacterial effects against *S. aureus* Good biocompatibility on fibroblasts	Improved wound closure, faster healing rate, reduced inflammation, promoted angiogenesis	[421]
PU/CA nanofibrous scaffolds incorporated with AgNPs-decorated GO and curcumin	Antibacterial effects against *P. aeruginosa* and *S. aureus* Enhanced cellular proliferation of fibroblasts	Improved neovascularization and collagen deposition, faster adnexal healing response, accelerated wound healing and advanced epidermis regeneration	[422]
PU foam dressings incorporated with AgNPs and asiaticoside	Antibacterial effects against *B. subtilis*, *E. coli*, *P. aeruginosa* and *S. aureus* Enhanced cellular proliferation of fibroblasts	Safe skin application, improved and accelerated wound healing	[423]

Abbreviations: PEO—polyethylene oxide; KGM—konjac glucomannan; MF—mafenide acetate.
